# Efficacy of supra-inguinal fascia iliaca compartment block in proximal femoral nail antirotation internal fixation for patients with intertrochanteric fractures

**DOI:** 10.12669/pjms.41.1.10715

**Published:** 2025-01

**Authors:** Genfeng Ding, Jinling Shang, Qing Wang

**Affiliations:** 1 Genfeng Ding Anesthesia Surgery Department, First people’s Hospital of Yong Kang, Yongkang, Zhejiang Province 321300, P.R. China; 2 Jinling Shang Tumor Radiotherapy Department, First people’s Hospital of Yong Kang, Yongkang, Zhejiang Province 321300, P.R. China; 3 Qing Wang Orthopedics Department 2, First people’s Hospital of Yong Kang, Yongkang, Zhejiang Province 321300, P.R. China

**Keywords:** Supra-inguinal fascia iliaca compartment block, Intertrochanteric fracture, General anesthesia

## Abstract

**Objective:**

To evaluate the efficacy of supra-inguinal fascia iliaca compartment block (S-FICB) in patients undergoing proximal femoral nail antirotation (PFNA) internal fixation surgery for intertrochanteric fracture (ITF).

**Methods:**

Retrospective analysis of 95 patients with ITF undergoing PFNA internal fixation surgery in the First People’s Hospital of Yong Kang from March 2021 to August 2023 was performed. Among them, 49 patients received general anesthesia (GA; GA group) and 46 patients received S-FICB combined with general anesthesia (S-FICB group). Mean arterial pressure (MAP), heart rate (HR), anesthesia effect, pain, stress index levels, functional recovery, and adverse reactions were compared between two groups.

**Results:**

Perioperative MAP and HR, extubation time, anesthesia recovery time, and dosage of remifentanil and propofol were significantly lower compared to the GA group (*P*<0.05). Perioperative pain level in the S-FICB group was significantly lower than in the GA group (*P*<0.05). On the first day after the surgery, stress response of the S-FICB group was significantly lower than that of the GA group (*P*<0.05). Patients who underwent S-FICB required significantly shorter time for getting out of bed and for straight leg elevation, and lower hospitalization time (*P*<0.05).

**Conclusions:**

Compared to GA, S-FICB for ITF internal fixation surgery is associated with more stable hemodynamics, lower pain levels, less consumption of opioids, lower perioperative stress response, reduced postoperative complications, and shorter hospital stay in patients during the perioperative period.

## INTRODUCTION

The trochanteric region of the femur is one of the areas most severely affected by bone loss.^1^ In elderly people, low bone mass and fragile bone structure significantly increase the incidence of fractures^2^ which are considered one of the main risk factors that endanger health and quality of life of elderly population.^2^,^3^ Treatment of intertrochanteric fracture (ITF) can be done conservatively or surgically.^1^,^3^ Conservative treatment methods require long-term bed rest and are associated with complications such as pressure ulcers, urinary tract infections, lung infections, and deep vein thrombosis. Even after fracture healing, the incidence of joint contracture and malunion in patients treated by the conservative approach remains high.^3^,^4^

Therefore, surgery is the preferred treatment for ITF, since internal fixation not only improves the biomechanical performance of patients with ITF, but also has the advantages of low surgical risk and reduced intraoperative bleeding.^2^–^5^ Studies have shown that internal fixation is associated with a favorable outcome in elderly and unstable patients.^2^,^4^,^5^ However, ITF mostly occurs in the elderly who have multiple underlying diseases and varying degrees of organ decline, making them less tolerant to traditional anesthesia approaches and opioids.^5^,^6^

Since the dosage of opioid drugs is directly linked to patients’ conditions, length of hospital stay, and unplanned admission,^6^ selection of a rational anesthesia in this population of patients is crucial. Supra-inguinal fascia iliaca compartment block (S-FICB) that is based on the injection of local anesthetic beneath the fascia iliaca, makes anesthetic drugs diffuse more easily, achieving good nerve block and postoperative analgesia.^7^ Studies have shown a promising efficiency of S-FICB in hip fracture surgery.^7^,^8^ However, the effectiveness of S-FICB in ITF internal fixation surgery is still unclear. This study aimed to evaluate the clinical efficacy of S-FICB in the surgical treatment of ITF from the perspectives of opioid dosage, hemodynamics, and functional recovery.

## METHODS

Clinical data of 95 ITF patients who underwent proximal femoral nail antirotation (PFNA) internal fixation surgery in the First People’s Hospital of Yong Kang from March 2021 to August 2023 were retrospectively selected. According to anesthesia records, patients receiving general anesthesia (GA) were included in the GA group, and patients receiving S-FICB combined with GA were included in the S-FICB group.

### Ethical Approval:

The ethical committee of our hospital approved our study with the number 202310311747000193131, on November 1^st^ 2023.

### Inclusion criteria:

- Patients with simple intertrochanteric fractures.
- Patients who have completed PFNA internal fixation.
- Age ≥ 60 years old.
- American Society of Anesthesiologists (ASA) classification is II-III.
- The clinical data was complete.

### Exclusion criteria:

- Patients with abnormal coagulation function.
- Patients with central nervous system and peripheral nervous system diseases.
- Patients with combined fractures of other parts or pathological or old fractures.
- Patients with preexisting limb dysfunction before the fracture.
- Patients with severe osteoporosis.
- Patients with combined cognitive impairment disorders such as Alzheimer’s disease and epilepsy.
- Patients with severe cardiovascular and cerebrovascular diseases.
- Patients with a history of alcohol and opioid abuse or dependence.
- Before the surgery, a routine venous channel was established for the patient and oxygen was administered through a mask.

### GA group:

Patients received tracheal intubation and intravenous injection of atracurium besylate (specification: 5ml/10mg; Hangzhou Hongyou Pharmaceutical Technology Co., Ltd.) 0.2mg/kg, sufentanil (specification: 1ml: 50μg; Yichang Renfu Pharmaceutical Co., Ltd.) 0.3-0.4μg/kg, etomidate 0.2mg/kg (specification: 10ml: 20mg; Jiangsu Enhua Pharmaceutical Co., Ltd.), propofol (specification: 20ml: 0.2g; Sichuan Guorui Pharmaceutical Co., Ltd.) 1mg/kg induction anesthesia, intraoperative remifentanil (specification: 1mg; Yichang Renfu Pharmaceutical Co., Ltd.), propofol Phenol maintenance anesthesia.

### S-FICB group:

Patients received S-FICB combined with GA. A high-frequency probe was placed on the affected side of the inguinal ligament using the M-Turbo ultrasound diagnostic instrument (Sono Sound from the United States), and the puncture position was determined under ultrasound guidance. A 10 cm puncture needle was inserted through the medial femoral approach, so that the needle tip reached the iliac fascia gap. After ultrasound-guided puncture of the iliac fascia, 30 mL (0.25%) of ropivacaine hydrochloride injection (specification: 75mg/10ml, Qilu Pharmaceutical Group) was administered. If the medication diffused within the gap, it indicated successful puncture and indwelling of the catheter. After completing S-FICB, GA with tracheal intubation was performed, and anesthesia induction, anesthesia maintenance drug dosage and usage, and BIS value settings were the same as those in the GA group. After the anesthesia took effect, internal fixation surgery was performed. The tracheal Intubation was removed after the patient woke up, was breathing, and had a stable circulation.

### Collection indicators:

- Baseline data, including gender, age, body mass index (BMI), ASA grade, surgery time, and underlying disease.
- Hemodynamic indicators, including mean arterial pressure (MAP) and heart rate (HR) five minutes before the anesthesia (T0), five minutes after the anesthesia (T1), at the time of skin incision (T2), and at the end of the surgery (T3).
- Anesthetic effects, including extubation time, anesthesia recovery time, consumption of remifentanil and propofol.
- Pain level, including pain levels before the anesthesia, and 12-, 24-, and 48 hours after the surgery. The visual analog scale (VAS) was used for evaluation, with a score range of 0-10 points. Higher the score indicated more severe pain.
- Stress index levels, including serum epinephrine (E), cortisol (Cor), and norepinephrine (NE) levels before and 1-day after the surgery.
- Functional recovery, including time to get out of bed, time to raise straight legs, and length of hospital stay.
- Adverse reactions such as drowsiness, nausea and vomiting, skin itching, limb numbness, and hypotension.

### Statistical analysis:

All data analyses were conducted using SPSS 26.0 software (IBM Corp, Armonk, NY, USA). The measurement data were represented by mean ± standard deviation, independent sample t-test was used for inter group comparison, and repeated measures analysis of variance was used for intra group comparison. Count data was analyzed using Chi square test to represent the number of cases. *P*<0.05 was statistically significant.

## RESULTS

**Fig.1:**
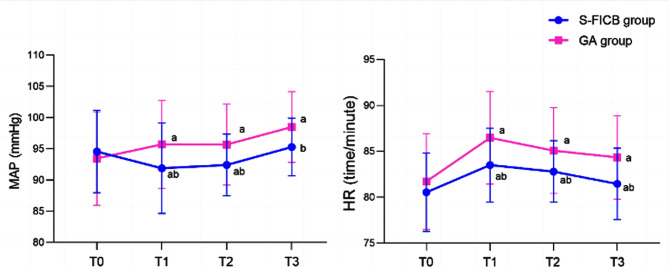
Comparison of MAP and HR levels between the two groups. Compared with T0 in the same group, ^a^*P*<0.05; Compared with the GA group, ^b^*P*<0.05; S-FICB: supra-inguinal fascia iliaca compartment block; GA: general anesthesia; MAP: mean arterial pressure; HR: heart rate; T0: before anesthesia; T1: five minutes after anesthesia; T2: at the time of skin incision; T3: at the end of surgery.

A total of 95 patients (49 males and 46 females) met the eligibility criteria for this study. Age of the patients ranged from 60 to 91 years, with an average age of 73.99 ± 7.90 years. There were 49 cases in the GA group and 46 cases in the S-FICB group, with no statistically significant difference in baseline data between the two groups (*P*>0.05) Table-I. There was no statistically significant difference in MAP and HR between the two groups at T0 (*P*>0.05). However, MAP and HR of the S-FICB group were significantly lower than those of the GA group at T1, T2, and T3 (*P*<0.05). Fig.1 Extubation time, anesthesia recovery time, consumption of remifentanil and propofol in the S-FICB group were significantly lower than those in the GA group (*P*<0.05) Table-II.

**Table-I:** Comparison of baseline data between two groups of patients.

Baseline data	S-FICB group (n=46)	GA group (n=49)	Statistical value	P value
Gender (male/female)	26/20	23/26	0.872	0.35
Age (years)	72.96±7.32	74.96±8.37	-1.238	0.219
BMI (kg/m^2^)	22.94±2.94	23.65±2.85	-1.191	0.237
ASA (II/III)	22/24	21/28	0.236	0.627
Operation time (min)	69.48±13.98	67.54±15.14	0.651	0.517
Perioperative bleeding (mL)	173.44±18.61	176.69±21.49	-0.788	0.433
Coronary heart disease (yes)	8 (17.39)	4 (8.16)	1.831	0.176
Hypertension (yes)	9 (19.57)	11 (22.45)	0.119	0.730
Diabetes (yes)	10 (21.74)	5 (10.20)	2.374	0.123

S-FCIB: supra-inguinal fascia iliaca compartment block; GA: general anesthesia; BMI: body mass index; ASA: American Association of Anesthesiologists.

**Table-II:** Comparison of anesthetic effects between the two groups.

Group	Extubation time (minute)	Anesthesia awakening time (minute)	Remifentanil(μg)	Propofol (mg)
S-FICB group (*n*=46)	16.87±4.36	17.41±4.10	424.8±63.75	527.96±87.39
GA group (*n*=49)	20.90±4.20	20.96±4.20	534.9±97.57	602.9±98.23
*t*	-4.587	-4.160	-6.549	-3.919
*P*	<0.001	<0.001	<0.001	<0.001

S-FCIB: supra-inguinal fascia iliaca compartment block; GA: general anesthesia.

**Fig.2:**
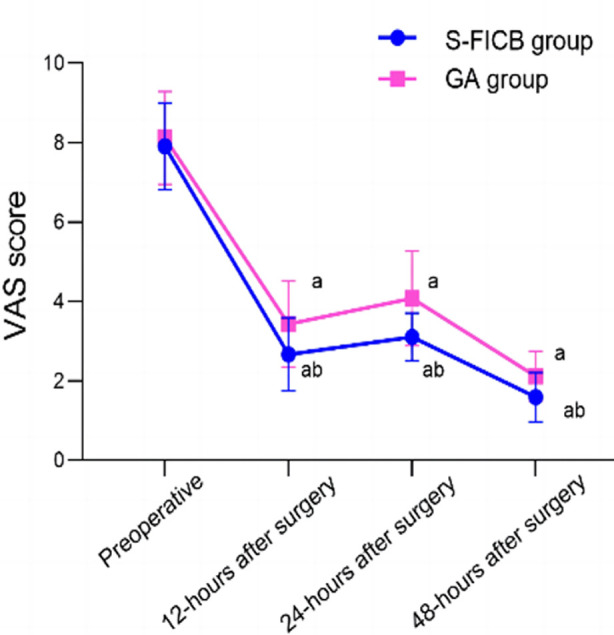
Comparison of VAS scores between two groups; Comparison with the same group preoperative, ^a^*P*<0.05; Compared with the GA group, ^b^*P*<0.05; **S-FICB:** supra-inguinal fascia iliaca compartment block; **GA:** general anesthesia; **VAS:** visual analog scale.

**Fig.3:**
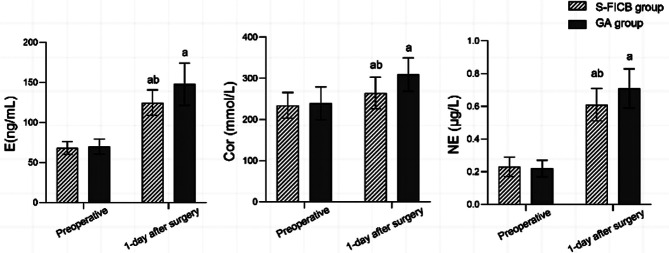
Comparison of stress indexes between the two groups; Comparison with the same group preoperative, ^a^*P*<0.05; Compared with the GA group, ^b^*P*<0.05; S-FICB: supra-inguinal fascia iliaca compartment block; GA: general anesthesia; E: epinephrine; Cor: cortisol; NE: norepinephrine.

There was no statistically significant difference in the VAS scores between the two groups before the anesthesia (*P*>0.05). The VAS scores of the S-FICB group were significantly lower than those of the GA group at 12, 24, and 48 hours after the anesthesia (*P*<0.05). Fig.2 Before the surgery, there was no statistically significant difference in the levels of E, Cor, and NE between the two groups (P>0.05). On postoperative Day One, levels of E, Cor, and NE in both groups were significantly higher than before the surgery, but were significantly lower in the S-FICB group compared to the GA group (*P*<0.05) Fig.3.

The time to get out of bed, straight leg elevation, and hospitalization in the S-FICB group were significantly shorter than those in the GA group (*P*<0.05). Table-III There was no statistically significant difference in the incidence of postoperative adverse reactions between the two groups of patients (*P*>0.05 Table-IV.

**Table-III:** Comparison of functional recovery indicators between the two groups.

Group	Bedtime (day)	Time to straight leg raise (day)	Hospital stays (day)
S-FICB group (n=46)	3.22±1.15	2.33±1.08	13.8±3.49
GA group (n=49)	3.92±1.11	2.98±1.03	16.02±2.3
*t*	-4.41	-3.023	-3.675
*P*	<0.001	0.003	0.002

S-FCIB: supra-inguinal fascia iliaca compartment block; GA: general anesthesia.

**Table-IV:** Comparison of adverse reactions between two groups.

Group	drowsiness	nausea and vomiting	pruritus	Limb numbness	hypotension	Total incidence rate (%)
S-FICB group (n=46)	2 (4.35)	0 (0.00)	1 (2.17)	0 (0.00)	1 (2.17)	4 (8.70)
GA group (n=49)	1 (2.04)	2 (4.08)	2 (4.08)	1 (2.04)	2 (4.08)	8 (16.32)
*χ* ^2^						1.252
*P*						0.263

S-FCIB: supra-inguinal fascia iliaca compartment block; GA: general anesthesia.

## DISCUSSION

S-FICB has been widely used in clinical practice as a regional anesthesia or analgesic method for lower limb surgeries such as knee, femur, and hip.^6^–^10^ The results of this study showed that S-FCIB combined with GA is associated with better outcomes in ITF patients undergoing surgical fracture repair. We have showed that MAP and HR of the patients in the S-FICB group were significantly lower than those of the patients who underwent the surgery under GA at T1, T2, and T3. S-FICB, therefore, was associated with more stable hemodynamics. These results are consistent with previous research.^9^,^10^ We may speculate that ITF patients experience strong pain during preoperative positioning and other manipulations, which exacerbates their discomfort and can easily cause significant fluctuations in vital signs, increasing the risk of cardiovascular and cerebrovascular accidents. Ultrasound-guided FICB anesthesia block that is performed before the patient is transferred onto the operating bed, has a good blocking effect on the obturator nerve, femoral nerve, lateral femoral cutaneous nerve, etc. It reduces the stimulation of related surgical operations on the patient’s body, allows to maintain stable circulation, and has a good analgesic effect.^9^,^10^

The results of this study also showed that S-FICB was associated with significantly lower extubation time, anesthesia recovery time, consumption of remifentanil and propofol. Our results suggest that S-FICB can achieve better anesthesia effects and reduce opioid consumption. It is plausible that the iliac fascia space has a wide range, and the use of multi-point needle puncture can simultaneously block lateral femoral cutaneous, femoral, and obturator nerves, which is more conducive to the better diffusion of local anesthetics, and markedly reduces the consumption of remifentanil and propofol.^11^,^12^ In agreement with our observations, the study by Bali C et al.^13^ showed that S-FICB technology has a significant opioid drug saving effect, thereby reducing opioid-related side effects in the first 24 hours after hip fracture surgery in elderly patients. Gola W et al.^14^ also showed that using FICB in total hip replacement surgery can reduce the demand for opioids, the number of complications, hospital stay, and ensure high patient satisfaction with the analgesic treatment used. The results of this study also support the above research conclusions.

In addition, this study showed that the VAS scores of the S-FICB group were significantly lower than those of the GA group at 12, 24, and 48h after the surgery. Our results are consistent with the study by Hao et al.,^15^ which showed that compared to conventional anesthesia, FICB has excellent analgesic effects during rest and exercise within one week after the surgery, and results in quick short-term restoration of hip joint function in postoperative elderly patients with hip fractures. This effect may be explained by the fact that S-FICB can evenly distribute local anesthetic drugs in the gaps of the iliac fascia through multi-point injection, effectively diffusing anesthetic drugs, blocking the conduction of the lateral femoral cutaneous nerve and femoral nerve, and achieving good analgesic effects.^14^,^15^

During the perioperative period, ITF patients experience sympathetic nervous system excitation, leading to the release of a large amount of E, NE, and Cor from the adrenal medulla and sympathetic nervous system to enhance the body’s ability to cope with nociceptive stimuli under stress.^16^,^17^ The results of this study showed that the postoperative levels of E, NE, and Cor in the S-FICB group were significantly lower than those in the GA group on the 1st day after the surgery, indicating that S-FICB can help alleviate stress responses. S-FICB accurately targets the location of the anesthesia block, and the needle tip is distanced far away from the femoral vein, femoral artery, and femoral nerve. Therefore, this method will not cause damage to the nerve, vital signs during surgery will remain stable, with no strong stress reaction.^14^–^17^

The results of this study have showed that functional recovery of patients, such as time to get out of bed, straight leg elevation, and hospitalization time were significantly shorter in the S-FICB group compared to the GA group. Although general anesthesia has a good muscle relaxation effect and meets surgical treatment needs, accompanying MAP and HR fluctuations often lead to a higher consumption of opioids and a more obvious perioperative stress response, leading to poor functional recovery in patients.^18^,^19^ S-FICB can accurately block the nerve tissue of the affected limb and adjust the direction of anesthesia diffusion under ultrasound guidance to determine the puncture position and path, which is beneficial for improving the analgesic effect, reducing the use of anesthesia drugs and the occurrence of adverse reactions. Moreover, postoperative pain is relatively mild, and patients are more willing to engage in passive and active movements on and under the bed early, ultimately promoting fracture healing.^15^–^17^

Our study has also showed no significant difference in the incidence of adverse reactions between the two groups. It has demonstrated that S-FICB is safe and reliable in ITF surgery. In agreement with our results, Yamamoto N et al.^20^ also indicated that compared to simple intravenous anesthesia, FICB improved postoperative motor pain without increasing the incidence of complications. However, Wang et al.^21^ found that compared to anterior lumbar muscle block, S-FICB was associated with significantly more incidences of quadriceps femoris weakness at two hours (P=0.008) and six hours (P=0.009) after the surgery. This discrepancy between the results may be related to the selection bias of sample size. Our study confirms that S-FICB has the advantages of convenient operation, significant analgesic effect, and is not associated with increased incidence of adverse reactions. This approach, therefore, may be considered method of choice as anesthesia for ITF patients.

### Limitations:

Firstly, it is a single center retrospective analysis with a small sample size and selection bias. Secondly, there has been a lack of extensive observation and analysis of adverse reactions such as postoperative respiratory depression, pulmonary infection, urinary tract infection, and deep vein thrombosis. Thirdly, the VAS score is greatly influenced by individual differences and subjective factors of patients, resulting in corresponding errors. Fourthly, the effectiveness of S-FICB may be influenced by human or technological factors. We will continue to conduct higher quality research in the future to validate the content of this conclusion.

## CONCLUSION

Compared with GA alone, S-FICB combined with GA can be safely and effectively used in ITF surgery, reducing perioperative pain, opioid consumption, perioperative stress response, postoperative complications, hospital stay, and improving patient quality of life during hospitalization.

### Authors’ contributions:

**GD:** Conceived, designed the study, and prepared the manuscript.

**JS** and **QW:** Collected the data, performed the analysis and Review.

All authors have read and approved the final manuscript and are is responsible for the integrity of the study.
